# The ever-lasting green tides: What can we do？

**DOI:** 10.1016/j.heliyon.2024.e25220

**Published:** 2024-01-28

**Authors:** Cheng-Gang Ren, Zhi-Hai Zhong, Zhi-Yi Liu, Shuang Lin, Yong-Kai Luo, Song Qin

**Affiliations:** a Yantai Institute of Coastal Zone Research, Chinese Academy of Sciences, 17 Chun-hui Road, Lai-shan District, Yantai, China; b Academician Workstation of Agricultural High-tech Industrial Area of the Yellow River Delta, National Center of Technology Innovation for Comprehensive Utilization of Saline-Alkali Land, Dongying, Shandong, China

**Keywords:** Green tides, Developed coastal area, Eutrophication

## Abstract

Macroalgal blooms (Green tides) are occurring more frequently in many regions of the world because of the combined effects of increasingly intense human activity and climate change. In the last decade, the world's largest *Ulva prolifera* green tide has become a recurrent phenomenon, appearing every summer in the southern Yellow Sea, China. Green tides can hurt coastal tourism and eradicate aquaculture and artisanal fishing. Eutrophication in nearshore waters is the ultimate explanation for the explosive growth of the macroalgal biomass, but the specific course of each nearshore green tide is often complex and requires in-depth and extensive research to develop effective mitigation strategies. Researchers have undertaken extensive studies on the prevention, control and mitigation of large-scale green algal blooms, and felicitated the utilization of green tide harmful biomass through bio-refining, bioconversion and other measures. However, due to the large-scale and *trans*-regional nature of the green tide, the government's administrative coordination measures are also essential for effective control. Nevertheless, it is becoming increasingly urgent to prevent and control the bloom at the early stage, and efficiently salvage and use these valuable raw materials.

Green tides have been recorded in many coastal areas worldwide, and an increasing number of reports have been published in the past three decades. Such macroalgal blooms are often directly associated with the intensified anthropogenic activity of coastal areas [1] (Fig. 1), which in turn are harmful to coastal human activities such as tourism and aquaculture.

**Fig. 1:**
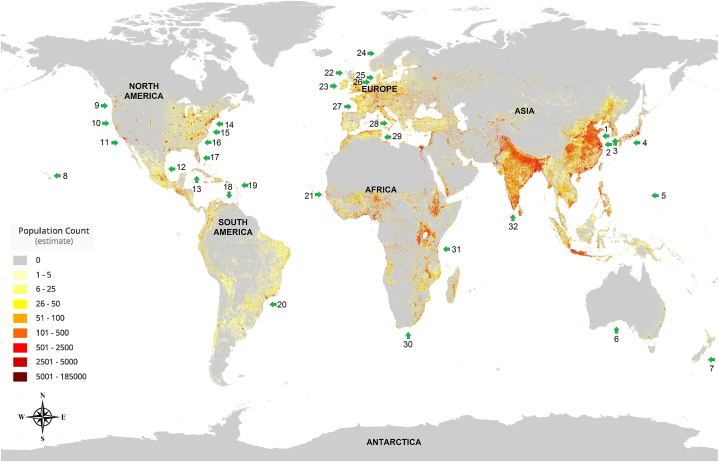
**Distribution of green tides worldwide since 1980.** The most frequently attacked coastal areas are densely populated areas. “1–2” marks the world's largest green tide. 1, China (Shandong); 2, China (Jiangsu); 3, Korea; 4, Japan; 5. USA (Guam Island); 6, Australia; 7, New-Zealand; 8, Hawaii; 9, USA (Washington); 10, USA (Oregon); 11, USA (California); 12, Mexico; 13, Cuba; 14, USA (Massachusetts); 15, USA (Connecticut); 16, USA (North Carolinas); 17, USA (Florida); 18, Venezuela; 19, Martinique; 20,Brazil; 21,Senegal; 22, Great-Britain; 23. Ireland; 24, Norway; 25, Denmark; 26, the Netherlands; 27, France; 28, Italy; 29, Tunisia; 30, South Africa; 31, Tanzania; 32, India. Population data is retrieved from the LandScan Program [16]. Green arrows show areas where green tides have broken out repeatedly. The color of the rectangle indicates the density of the population. All labeled areas are according to reports in peer-reviewed papers or issued by government agencies with credibility. (For interpretation of the references to color in this figure legend, the reader is referred to the Web version of this article.)

Macroalgae (Chlorophyta, green algae; Phaeophyta, brown algae; and Rhodophyta, red algae) have important roles in marine ecosystems as food and habitat for animals. However, excess macroalgal abundance can have negative impacts on the environment by decreasing species richness and abundance, especially of seagrasses and fish [[1], [2], [3]]. Rapid industrialization in coastal zones cause eutrophication, which promotes macroalgal growth and reproduction [1]. For example, increased nitric oxide production due to altered marine nutrient levels provides conditions for sporulation of the green alga *Ulva*, which is the most reported and spatially distributed blooming algae, and causes the largest green tide blooms in the Yellow Sea [1,2]. *Ulva* spores attach to substrates (such as laver culture rafts and parent *Ulva* thalli), which then develop into new thalli and produce more spores. As these mats grow, they hinder air–gas exchange, which can cause hypoxia in relatively enclosed sea areas (such as bays), and prevent sunlight from passing into the sea, which kills phytoplankton that are essential for marine food webs [1].

Since 2000, the frequency and size of green tides have dramatically increased worldwide [20]. Such events have been reported in the United States, United Kingdom, France, Italy, Denmark, Japan, and other countries [[20], [21], [22], [23]] (Fig. 1). There are substantial economic losses associated with macroalgal blooms, such as impacts on tourism and the local economy. For example, a large-scale green tide was first reported off the coast of Qingdao, China in 2007. In 2008, an unprecedented green tide hit the same area again, severely disrupting the Olympic sailing events planned in the same area. The green tides recorded during this period were the world's largest algal blooms at the time, both in the area of sea affected and in the biomass produced [4]. Since then, although the coverage area varied greatly from year to year, the green tide has become a recurring phenomenon in the Southern Yellow Sea [5].

Most green tide events occur in closed or semi-closed bays or estuaries and algal blooms are generally native species because eutrophic seawater in such areas cannot be effectively dissipated to the ocean in a short amount of time [13]. For the Yellow Sea green tides, the scenario is seemly different, but there are intrinsic similarities with other green tide outbreaks. Apart from its spectacular size, the most remarkable feature of the Yellow Sea green tide is the long-distance drift (>500 km) of the seaweed mat. The outbreak began in the coastal area of Jiangsu Province in late April and spread northeast with wind and surface flow, reaching Shandong Peninsula in June and July; then, it disappeared because of precipitation and decomposition in late July [14].

Similar to other green tide outbreaks, the Yellow Sea green tide occurs in a large closed environment formed by a combination of specific hydrological features and regional biogeochemical processes. The southern Yellow Sea is a semi-closed continental sea in the northwest Pacific Ocean surrounded by Shandong Peninsula, northern Jiangsu Province, and the Korean Peninsula. This region has specific topographic, hydrological, chemical, and ecological characteristics [15]. In addition, the southern Yellow Sea is strongly controlled by the East Asian monsoon climate, with north winds prevailing in winter and south winds prevailing in summer. In the coastal area of the southern Yellow Sea, the seabed topography significantly changes and the current structure is complex. Because of the influence of the Yellow Sea Warm Current [16] and the Yellow Sea Cold Water Mass [17] at the bottom in summer, a significant front can be formed in the confluence area of coastal water and near sea water [18]. Previous studies noted that a tide-induced upwelling system off the northern shoal in summer could provide a sustainable supply of nutrients for the growth of *U. prolifera* drifting on the surface of the southern Yellow Sea [19]. In addition, Bao et al. (2015) used satellite data and a numerical model (FVCOM) to study the drift trajectory of green algae under tidal and surface winds, and found that wind became the dominant drift force of floating green algae in the northern Jiangsu shoal. This is consistent with satellite tracking of northward *U. prolifera* movement [12,14] and cruise observations [20].

Adjacent to the southern Yellow Sea, this green tide has also occurred in Shandong, Jiangsu, and Shanghai, which are highly developed areas that are pivotal components of China's coastal industrial and marine economy. Consequently, the damage caused by this green tide is incalculable. Since 2008, Qingdao, a coastal city in Shandong Province, has invested more than USD200 million to remove a total of 35.61 million tons of *U. prolifera* invading the coastline (http://ocean.qingdao.gov.cn). The 2009 green tide caused direct economic losses of nearly USD100 million to Shandong Province (http://www.gov.cn/bumenfuwu/soa). Because of the intensity and increasingly serious reality of the *U. prolifera* green tide outbreak in the Yellow Sea and the consequent economic losses and social impacts, this green tide cannot be ignored.

Consequently, prevention and mitigation measures are being developed to reduce the negative environmental and economic impacts of these blooms, and strategies have been developed to utilize macroalgal biomass for commercially valuable purposes. There are several approaches currently used to monitor and predict harmful algal blooms, such as *in situ* monitoring of fish and shellfish, satellite remote sensing to detect increases in blooms, and predictive modeling to facilitate immediate responses (e.g., harvesting or deployment of mitigation measures). However, current models are better at predicting harmful algal bloom initiation than termination, and many over-predict duration [3].

Current control measures include biological, physical, and chemical approaches. Restoration of coastal habitats with seagrass with algicidal bacteria which capable of controlling algal growth through physical association or the production of algicidal compounds, or seaweed that secretes algicidal chemicals is a biological approach to controlling these blooms. Physical (e.g., barriers, or removal by water column mixing, filtering, flocculation, sediment burial, and dredging, or cell lysis using ultrasound) and chemical control methods (e.g., natural biocides, biosurfactants which produced at the microbial cell surface, and reduce surface and interfacial tension and allelochemicals which produced by organisms of one species that modify the behavior of organisms of a different species, synthetic chemicals, isolated algicidal compounds, or metallic compounds) can be used, but are often more costly, lack specificity to the bloom species, and are often less effective in coastal environments [3,6]. Consequently, additional biological approaches should be developed [6].

It has also been proposed that we may be able to reduce the environmental impacts of macroalgal blooms and adopt a circular economy by turning nuisance algae into useful and commercially viable products [2]. Macroalgae are promising agents for biofuels, chemicals (e.g., glycerol and organic acids), food additives, and bioactive compounds because of their chemical composition, the global abundances, and knowledge regarding their growth and occurrence patterns [7]. Although microalgae have a fast growth rate and high lipid content, macroalgae have higher cost effectiveness (e.g., because of higher productivity) [8]. Unlike terrestrial crops used for biofuel production, macroalgae do not compete with food production by requiring farmland, freshwater, and fertilizers [7]. Moreover, they have diverse polysaccharides, which makes them promising sources of food, cosmetics, and pharmaceuticals [2]. For example, biorefining of Phaeophyta, such as *Sargassum* spp., is beneficial because they can produce carotenoids, phenolic compounds, alginate fractions, and antioxidant, antifouling, antimicrobial, and antitumor compounds [2]. Additionally, Chlorophyta are ideal for use in biorefinery because of their high biomass, protein content, proportions of sugars, and diversity of essential amino acids; however, they are the least researched with regard to bioprocessing activities [2].

Since 2005, there has been increasing research on the utilization of macroalgae worldwide, with the majority focusing on Phaetophyta and somewhat less addressing Chlorophyta and Rhodophyta. Biorefining to produce multiple products has been the most researched application of macroalgae, but feasibility assessments indicated that biofuel production may be a more effective use of macroalgae [2]. In China, there have been few mitigation options applied, and macroalgae have been primarily used as fertilizer ingredients [1]. However, the growth rates and net primary productivity of macroalgae, such as *Ulva*, is often higher than that of some terrestrial plants and crops that are typically used as feedstocks for biorefineries [9,10]. Therefore, macroalgae show promise as economically valuable resources (Fig. 2).

**Fig. 2:**
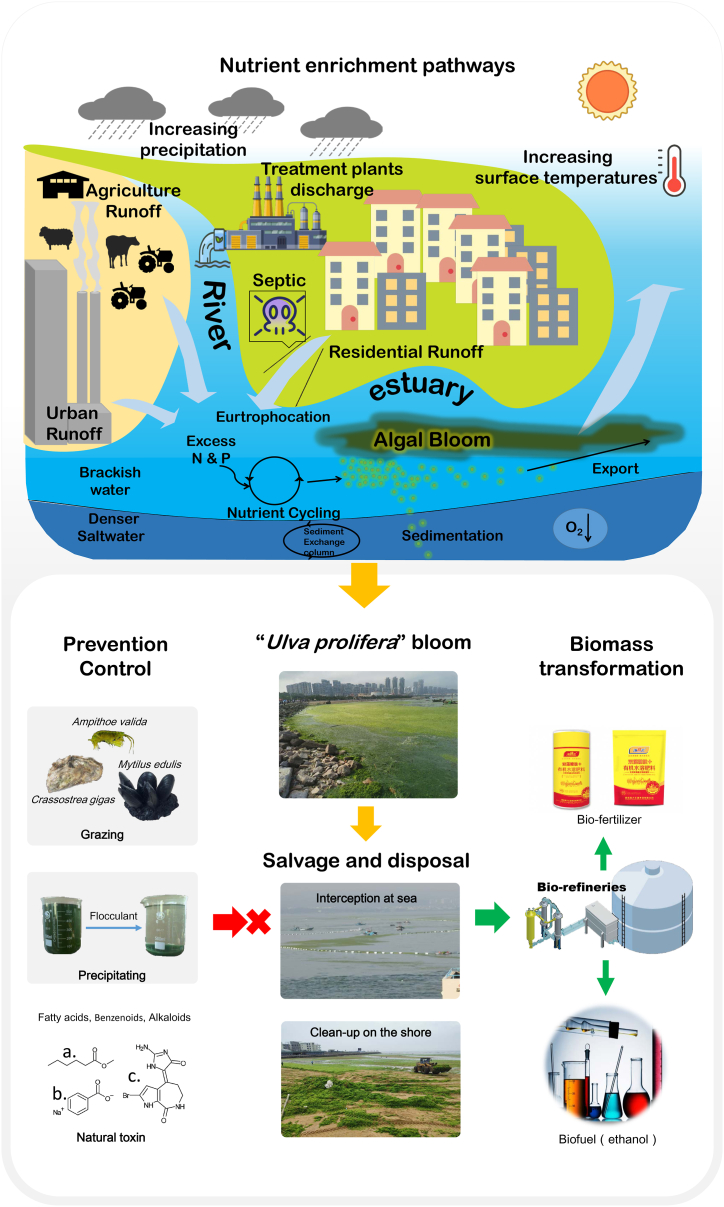
**Illustration of the formation, prevention, treatment, and utilization strategies of the *Ulva prolifera* bloom.** Nutrient enrichment pathways are shown via river runoff, storm water runoff (Urban Runoff and Residential Runoff), and atmospheric precipitation and demonstrate the effects of anthropogenic nutrient enrichment on *Ulva* (*Enteromorpha*) *prolifera* bloom and associated consequences, e.g., oxygen depletion of bottom waters (O_2_↓). a. Methyl hexanoate is used for *U. pertusa* spore settlement [17]; b. sodium benzoate is used for *U. prolifera* antifouling [18]; and c. hymenialdisine is used for *U. prolifera* spore settlement [19]. In 2008, a fertilizer production line was built in China that can process 10,000 metric tons fresh *U. prolifera* per day (BMSG™, www.bmsg.com).

One way macroalgae is economically valuable is by its use in wastewater treatment. Most nitrate wastewater treatment methods that target denitrification are expensive and involve nitrate dissipation. However, introducing treated wastewater with abundant nitrates into *Ulva* cultivation pools facilitates *Ulva* growth and reproduction, and *Ulva* can then be harvested as feedstock for biofuel production [1]. Such macroalgal biomass is more affordable yet shares similarly high heavy metal removal efficiency compared with expensive synthetic adsorbents [11].

Residual and waste biomass can also be used to simultaneously produce economically useful materials and dispose of undesirable biomass [7]. However, most studies have focused on wild non-blooming, or cultivated materials; few have examined the use of macroalgal blooms [2]. Macroalgal blooms can be problematic because they often include several species that require different treatments based on compositional characteristics and desired products [2]. Therefore, it can be hard to predict yield and price from derived products, especially because yield highly depends on pre-treatment and processing procedures. Moreover, there are often uncertainties in biomass yield, harvesting, and transportation costs; for bloom utilization to be a viable approach, the costs must be considerably lower than that of cultivated seaweed biomass [7].

To date, most research on macroalgal biomass use has focused on renewable energy sources. Current fossil fuel reserves are insufficient for the increasing demand of the expanding human population and will become depleted [10,12]. Production of biofuels (bio-gas, bioethanol, and crude oils) by macroalgae is potentially an environmentally sustainable option [1,12]. Replacing fossil fuels with marine energy renewable energy can decrease harm to air and water quality, supply chain and transportation risks, and operational costs [13]. Moreover, macroalgae represent a good potential non-consumable biofuel source because they are considered safe and they can exponentially grow in saline water and adverse conditions [12].

It was reported that biomethane recovery from macroalgae (such as the genera *Sargassum*, *Gracilaria*, *Laminaria*, *Ascophyllum*, and *Ulva*) is comparable to yields from terrestrial energy crops, with 50 %–60 % biomethane yields [7]. In recent years, there have also been new bioreactor designs developed to improve the bioconversion of seaweed to biomethane (e.g, solid-concentrating vertical and baffle flow reactors, and a fluidized bed reactor) to address previous limitations in loading rates and system performance [7]. Nanotechnology has also recently been implemented to enhance biofuel generation [12]. Although the economic viability and sustainability of these processes are still unclear, the development of large-scale biorefineries would have societal benefits (e.g., energy security and economic development through employment) [12].

A representative example of a harmful algal bloom that needs to be controlled is the *U. prolifera* green tide in the Yellow Sea, and the currently known cause, prevention, treatment, and utilization strategies of this bloom are shown in the Fig. 2. Normally, the first approach for controlling green tides should be to remove the algal mass from the sea and shoreline; if such measures are not implemented before the algae decay, the sea pollution problem will become a land pollution problem. For example, at St. Brieuc, France, coastal pollution was converted to land-based pollution at the time of dumping [14]. To resolve this issue, the raw materials should be pre-treated as quickly as possible. Later on, they can be composted, dehydrated and made into feed for poultry and aquaculture, processed into food, and biorefined into fuel [14,15]. However, to date, the most commercially viable approach is to use it as fertilizer. In 2008, a Chinese company built a fertilizer production line capable of processing 10,000 tons of fresh *U. prolifera* per day, and they have produced and sold dozens of products worldwide (BMSG™, www.bmsg.com) (Fig. 2).

As a matter of fact, in the current landscape of limited per capita resources and a declining ecological environment, macroalgae emerge as a distinctive raw material capable of replacing unsustainable fossil resources. Offering a versatile range of applications, macroalgae serve as valuable inputs for biorefineries, facilitating the production of fuels, chemicals, food ingredients, and pharmaceuticals [24]. This underscores its potential significance in shaping the future bioeconomy. The Ulvaceae family within the Chlorophyta group is particularly noteworthy for its contribution to green tide outbreaks, with notable members including *U. prolifera*, *U. linza*, *U. compressa*, *U. intestinalis*, and *U. clathrata*. Across Asia, Ulvaceae finds widespread utilization in diverse sectors, serving as a promising candidate for both food and medicinal purposes. *U. prolifera*, for instance, boasts substantial quantities of sulphate polysaccharides (SPs) and other bioactive substances [[25], [26], [27], [28], [29]].

Biorefining stands out as the process wherein raw biomass undergoes conversion into a myriad of products, encompassing food, biomaterials, and biofuels. A key tenet of biorefining lies in its commitment to sustainability, demanding economic viability while adhering to zero waste and minimal environmental impact principles. The integrated refining of macroalgae is anticipated to support the production of high-value goods and biofuels [24]. Notably, macroalgae, including Chlorophyta, exhibit considerably higher growth rates compared to traditional land crops. For instance, *U. compressa* displays a biomass accumulation efficiency of 1460 g C m−2 years−1, surpassing the 631 g C m^−2^ years ^−1^ for rice [30]. Furthermore, Chlorophyta showcases resilience to varying seawater conditions, including salinity, irradiance, and temperature, yielding unique structural molecules like SPs. Its value is further augmented by a high soluble dietary fiber content (up to 55 % in macroalgae), top-tier protein quality, and markedly superior free radical scavenging properties compared to land crops [31].

The distinctive advantages of Chlorophyta position it as a promising bio-refining material. Firstly, Chlorophyta can be transformed into diverse food additives due to their rich composition, encompassing proteins (lectin and taurine), fibers (SPs and ulvan), vitamins (tocol), antioxidants (carotenoids and chlorophyll), essential amino acids, polyunsaturated fatty acids, and minerals [[32], [33], [34]]. Secondly, these organisms find application as animal and fish feed [33]. Beyond that, ongoing research explores broader applications such as energy and biofuel production [35], bioremediation, and water treatment, leveraging Chlorophyta's remarkable capacity to absorb and accumulate nutrients and metals [36]. Polysaccharides derived from Chlorophyta prove to be excellent biomaterials with versatile applications, ranging from tissue engineering to papermaking [37]. In agriculture, Chlorophyta extracts serve as plant biostimulants [38], while their pigments are being investigated for bioelectronic applications like solar cells [39].

The maximization of biomass benefits is already achievable by concurrently producing two or more products using Chlorophyta [40,41]. Gajaria et al. [42] demonstrated an integrated cascade biorefinery process using *U. lactuca* as raw materials, extracting five chemical products: minerals, lipids, ulvan, proteins, and cellulose. Notably, the yield of products in this comprehensive sequence closely aligns with that of products extracted individually, indicating that the co-extraction of various products does not significantly compromise product yield in the integrated biorefinery process. Additionally, the consumption of chemical solvents in the cascade biorefinery is reduced by approximately 28 %–39 %, thereby mitigating the economic and environmental costs associated with biomass processing [43]. Consequently, the integrated sequential extraction method stands as a valuable approach to fully harness the biorefinery potential of macroalgae.

Although researchers have explored the prevention, control, and mitigation of macroalgal blooms, administrative measures by the government are also essential. First, governments and society must take the threat of these blooms seriously and increase research funding. For example, to date, China's National Research Fund has launched several green tide-related projects. These comprehensive, multidisciplinary, and cross-border initiatives will contribute to a better understanding of the mechanism underlying green tide occurrence in the context of specific hydrological characteristics and regional biogeochemical processes, and the impact of human industrial and agricultural activities [15]. Another example is that legislation to reduce the flow of chemicals and nutrients into the ocean has been shown to be an effective means of reducing water eutrophication in the USA (www.boem.gov). Second, the government should clarify the relationship between responsible parties to improve the legal and regulatory systems; encourage researchers, enterprises, and local residents to create a favorable social environment; and actively participate in macroalgal bloom management. However, because *U. prolifera* in the Yellow Sea involves different local governments, whether there is unified and coordinated prevention and control measures among them will ultimately affect the ability to control and respond to *U. prolifera* outbreaks.

Green tides manifest in numerous temperate coastal regions worldwide. The most prevalent species associated with these occurrences is Ulva. For instance, the green tide in the Yellow Sea, China, has garnered global attention since 2008. This surge has inflicted substantial economic losses on local mariculture, tourism, and the environment. Nevertheless, the algae biomass responsible for green tides is also recognized as a natural asset that can be harnessed for producing organic fertilizers and fuels, among other applications. As global climate change intensifies, the prevalence of ecological imbalances such as green tides is expected to worsen. Consequently, there is an urgent call for those in relevant areas to formulate strategies for preventing and controlling green tides. Looking ahead, the optimal approach to address large algae outbreaks, including green algae, involves early-stage prevention and control of biomass, subsequently reducing it. Furthermore, utilizing biomass resources can generate economic benefits from uncontrollable biomass.

## Data availability statement

No data was used for the research described in the article.

## Funding

This work was financed by The National Key Research and Development Program of China, 2022YFC3106001;10.13039/501100007129Shandong Natural Science Foundation, ZR2021MC106; Academician Workstation of Agricultural High-tech Industrial Area of the Yellow River Delta, National Center of Technology Innovation for Comprehensive Utilization of Saline-Alkali Land, Dongying, Shandong, China and Science & Technology Specific Projects in Agricultural High-tech Industrial Demonstration Area of the Yellow River Delta, Grant No: 2022SZX12, Innovation/Entrepreneurship Project of Shandong Green Industry and Environmental Security Innovation and Entrepreneurship Community, Grant No. 2023-LSGTT-CX-004.

## CRediT authorship contribution statement

**Cheng-Gang Ren:** Writing – original draft, Investigation, Funding acquisition, Conceptualization. **Zhi-Hai Zhong:** Resources, Funding acquisition. **Zhi-Yi Liu:** Writing – review & editing, Validation. **Shuang Lin:** Writing – review & editing. **Yong-Kai Luo:** Writing – review & editing. **Song Qin:** Writing – review & editing, Conceptualization.

## Declaration of competing interest

The authors declare the following financial interests/personal relationships which may be considered as potential competing interests:Cheng-Gang Ren reports administrative support was provided by Yantai Institute of Coastal Zone Research. Cheng-Gang Ren reports a relationship with Yantai Institute of Coastal Zone Research that includes: employment.
